# Hypertension and traditional risk factors for cardiovascular diseases among treatment naïve HIV- infected adults initiating antiretroviral therapy in Urban Tanzania

**DOI:** 10.1186/s12872-023-03332-6

**Published:** 2023-06-20

**Authors:** Tosi M. Mwakyandile, Grace A. Shayo, Philip G. Sasi, Ferdinand M. Mugusi, Godfrey Barabona, Takamasa Ueno, Eligius F. Lyamuya

**Affiliations:** 1 Department of Clinical Pharmacology, School of Biomedical Sciences, Campus College of Medicine, Muhimbili University of Health and Allied Sciences (MUHAS), Dar es Salaam, Tanzania; 2 Department of Internal Medicine, School of Clinical Medicine, Campus College of Medicine, Muhimbili University of Health and Allied Sciences (MUHAS), Dar es Salaam, Tanzania; 3 Division of Infection and Immunity, Joint Research Centre for Human Retrovirus Infection, Kumamoto University, Kumamoto, Japan; 4 Collaboration Unit for Infection, Joint Research Centre for Human Retrovirus Infection, Kumamoto University, Kumamoto, Japan; 5 Department of Microbiology and Immunology, School of Diagnostic Medicine, Campus College of Medicine, Muhimbili University of Health and Allied Sciences (MUHAS), Dar es Salaam, Tanzania

**Keywords:** Dyslipidaemia, Obesity, Hypertension, Diabetes mellitus, Cardiovascular diseases, HIV, Antiretroviral therapy, Tanzania

## Abstract

**Background:**

Cardiovascular diseases (CVDs) have become an important cause of ill health and death among people living with HIV and/or AIDS (PLHIV) in the antiretroviral therapy (ART) era. There is scarce data on the burden of hypertension (HTN) and risk factors for CVDs among PLHIV in developing countries, including Tanzania during the ART era.

**Objective(s):**

To determine the prevalence of HTN and risk factors for CVDs among ART naïve PLHIV initiating ART.

**Methods:**

We analysed baseline data of 430 clinical trial participants on the effect of low-dose aspirin on HIV disease progression among HIV-infected individuals initiating ART. HTN was the outcome CVD. Traditional risk factors for CVDs studied were age, alcohol consumption, cigarette smoking, individual and family history of CVDs, diabetes mellitus (DM), obesity/overweight, and dyslipidaemia. A generalized linear model (robust Poisson regression) was used to determine the predictors for HTN.

**Results:**

The median (IQR) age was 37 (28, 45) years. Females were the majority contributing 64.9% of all participants. The prevalence of HTN was 24.8%. The most prevalent risk factors for CVDs were dyslipidaemia (88.3%), alcohol consumption (49.3%), and overweight or obesity (29.1%). Being overweight or obese predicted the occurrence of HTN, aPR 1.60 (95% CI 1.16–2.21) while WHO HIV clinical stage 3 was protective against HTN, aPR 0.42(95% CI 0.18–0.97).

**Conclusion:**

The prevalence of HTN and traditional risk factors for CVDs in the treatment naïve PLHIV initiating ART are significant. Identifying these risk factors and managing them at the time of ART initiation may lower future CVDs among PLHIV.

**Supplementary Information:**

The online version contains supplementary material available at 10.1186/s12872-023-03332-6.

## Introduction

Over the last decade, most regions of the world including Sub-Saharan Africa (SSA) have experienced significant declines in both new HIV infections and AIDS-related deaths. This decline has been attributed to the introduction and increasing coverage of antiretroviral therapy (ART) [1–7]. While AIDS-related deaths are declining, incidences of non-AIDS complications such as cardiovascular diseases (CVDs) are increasingly becoming important causes of morbidity and mortality in people living with HIV and/or AIDS (PLHIV) [6]. For instance, hypertension (HTN) as a CVD itself and a risk factor for many other CVDs has been showing an upward trend among PLHIV like that in the general population. In fact, some studies have reported a higher prevalence of HTN in PLHIV than in the general population [8–10]. HTN has recently been reported to significantly contribute to cardiovascular (CV) morbidity and mortality among PLHIV. Indeed, adult PLHIV with HTN are at more risk for CV events and all-cause mortality than HIV- uninfected hypertensive adults [11–14]. For instance, the risk for incident acute myocardial infarction (AMI) is twice higher in PLHIV with HTN than in the general population with HTN [11].

Regional differences in the prevalence of HTN among PLHIV have been observed. In Africa, the prevalence of HTN among PLHIV ranges from 6% to as high as 50% in ART-treated and slightly lower (2–41%) among ART naïve patients. This may mean that ART has a role in the pathophysiology of HTN in PLHIV.

Despite the possible role of adverse effects of ART in increasing the risk for HTN in PLHIV, the pathophysiology of HIV-associated HTN and other CVDs is poorly understood and complex involving the interplay of many other factors such as the HIV virus itself, improved longevity, chronic inflammation, persistent immune activation, and traditional risk factors for CVDs [15–20].

The traditional risk factors for CVDs include HTN, overweight and obesity, dyslipidaemia, diabetes mellitus (DM), cigarette smoking, and a family history of CVDs, among others [21]. Additionally, in a recent review, hyperglycaemia even in non-diabetics has been shown to be associated with CV events. In fact, strict glycaemic control offers cardioprotective benefits during acute CV events [22]. Recent studies have reported that the traditional risk factors for CVDs are more prevalent and have a much greater impact on PLHIV than the general population [23]. Therefore, screening for these risk factors for their early detection and timely management is essential for the prevention and treatment of CVDs in PLHIV. In addition to optimal ART offered at the HIV care and treatment centres (CTCs), the world health organization (WHO) currently recommends that PLHIV should receive appropriate interventions such as smoking cessation, blood pressure (BP) control, lipid-lowering therapy, glucose control, weight management, and physical activity, to decrease their risk for CVDs [24].

In Africa including Tanzania, data on the prevalence of HTN and the traditional risk factors for CVDs among PLHIV are scarce. The availability of such data is critical for successful implementation of HIV and non-communicable diseases integrated care for PLHIV. Studying the magnitude of HTN and the associated traditional risk factors for CVDs in PLHIV prior to ART initiation may help to inform a strategy to identify those individuals at risk for CVDs and offer appropriate intervention to reduce their risk. Importantly, this may help monitor the long-term impact of the default first-line ART regimen in use, on the risk for CVDs and therefore, adjust the choice of regimen accordingly. In this study, we have comprehensively analysed the traditional risk factors for CVDs and determined their association with HTN among PLHIV in urban Tanzania prior to ART initiation.

## Methods

### Study design, study setting, study population

This article represents a report of a cross-sectional analysis of baseline data participants in a double-blind parallel-group randomized placebo-controlled phase 2 A clinical trial. The trial aimed at assessing the effect of low-dose aspirin on HIV infection among HIV-infected individuals initiating ART. The trial was registered both in the WHO recognized Pan African Clinical Trial Registry (PACTR) in Africa with registration number PACTR202003522049711 and in the US based ClinicalTrials.gov with registration number NCT05525156. A detailed methodology for the trial has been published elsewhere [25]. In brief, the trial recruited participants from three care and treatment centres (CTCs) located in three different hospitals in Dar es Salaam, Tanzania: Mbagala Rangi tatu hospital (MRTH), Temeke (TRRH) and Mwananyamala (MRRH) regional referral hospitals beginning in March 2020 to June 2022. The trial had to suspend recruitment for three months owing to the COVID-19 pandemic thus the last participant follow-up was in June 2022. The three participating clinics are large-volume CTCs supported by the Management and Development for Health (MDH) Organization and operate six days a week. A total of three to five newly HIV-infected individuals are registered at each CTC per day. The inclusion criteria into the trial were: being newly recruited or diagnosed PLHIV, consenting, ART naïve, aged 18 years or older, starting ART at the time of recruitment into the trial, and willing to remain in the study for six consecutive months. ART naivety was first assessed by self-reporting by the participants followed by confirmation of naivety from the program ART registry. The exclusion criteria were: being asthmatic, being pregnant, predisposed to bleeding, on antithrombotic therapy or therapy with trial-prohibited drugs (supplementary file 1), having active or history of peptic ulcer disease, having previous intolerance or allergy to aspirin or any aspirin-containing products and/or having severe renal disease (estimated glomerular filtration rate < 30 mil/min/1.73 m2). The trial consecutively enrolled 430 participants who fulfilled all the inclusion and none of the exclusion criteria.

### Data collection

Eligible participants underwent interviews and physical examinations and had their sociodemographic and clinical data recorded. Factors such as age, alcohol consumption, cigarette smoking, individual and family history of CVDs and DM were recorded. Drugs used by participants e.g., antihypertensives, antidiabetics, and antidyslipidaemics were recorded. For each participant, two BP readings were recorded using a digital sphygmomanometer (Yuwell YE660D, Jiangsu Province, China) from the left arm in a seated position, five to ten minutes apart. The mean of the two BP readings was then calculated and used as an individual’s BP [26]. Body weight in kilograms was taken with a participant on minimum clothing using a digital weighing scale (Health O Meter, 500KL, China); and body height in centimetres was measured with the participant wearing no shoes using a stadiometer (Health O Meter, 500KL, China). Body mass index (BMI) was then calculated as a quotient of weight in kilograms and squared height in metres (kg/m^2^) [27]. The participants also provided 4 mL of non-fasting [28] antecubital venous blood samples in an empty sterile red-topped vacutainer tube that were sent in a cool box at room temperature, to an off-site accredited research laboratory within 6 h since collection for processing and storage before analysis. Upon arrival at the laboratory, the blood samples were immediately centrifuged at 2500 rpm to obtain sera. The sera were then consecutively stored at -80° Celsius from March 2020 until analyses, in December 2022, of the lipid profile: total cholesterol (TC), high-density lipoprotein cholesterol (HDL-C), and triglycerides (TG) using the COBAS Integra 400 Plus (Roche Instruments Centre AG, Rotkreuz, Switzerland) analyser. Low-density lipoprotein cholesterol (LDL-C) was estimated using the Friedewald equation [29].

### Definitions of HTN and risk factors for CVDs

Pre-HTN was defined as mean systolic blood pressure (SBP) of 120–139 mmHg and/or mean diastolic blood pressure (DBP) of 80–89 mmHg [30]. HTN was defined as current or past use of antihypertensives and/or mean SBP ≥140 mmHg and/or mean DBP ≥ 90 mmHg and/ or history of HTN(excluding the history of pregnancy-related HTN) [30]. Risky age for CVDs was defined as 45 years or older for male participants and 55 years or older for female participants [31]. Alcohol consumption was defined as the current or history of regular consumption of alcohol. Cigarette smoking was defined as the current or history of regular cigarette smoking. DM was defined as a history of DM and/or current or past use of antidiabetics. The history of CVDs was defined by the participant’s previous history of stroke and/or myocardial infarction (MI) [31]. Family history of CVDs was defined as the occurrence of hypertension, and/or stroke, and/or MI in participants’ first-degree relatives [31]. Overweight and Obesity were defined as a BMI of 25.0 to 29.9 kg/m^2^ and ≥ 30.0 kg/m^2^, respectively. Dyslipidaemia was defined as non-fasting serum TC ≥5.17 mmol/ L and/or LDL-C ≥ 3.36 mmol/ L and/or TG ≥ 1.70 mmol/ L and/or current use of antidyslipidaemics regardless of sex and/or HDL-C <1.03 mmol/ L for men or HDL-C <1.29 mmol/ L< for women [31].

### Data management and statistical analysis

Data were recorded in case report forms (CRFs) and double entered, verified and cleaned in a password-protected computer before being analysed using statistical software for social sciences (SPSS) for windows version 26 (Inc., Chicago, Illinois). A comparison of the data to the source data was done to ensure accuracy and completeness.

Descriptive statistics were used to describe different characteristics of study participants. Continuous variables were presented as mean ± standard deviation (SD) or median (interquartile range (IQR)) depending on the normality of the data. Categorical variables were expressed as frequencies and percentages. Univariable and multivariable analyses were carried out using a generalized linear model (robust Poisson regression) to examine the predictors for HTN and other CVDs. The variables that had a p-value < 0.2 in the univariable analysis were included in the multivariable analysis. A p-value of 0.05 in the multivariable analysis was considered statistically significant.

## Results

### Sociodemographic and clinical characteristics

The socio-demographic and clinical characteristics of participants are presented in Table 1. A total of 430 treatment naïve HIV-infected individuals initiating ART were enrolled. The majority were female contributing 64.9% of all participants. The median (IQR) age was 37 (28, 45) years. The MRTH site contributed the most participants (61.4%). The majority of the participants had attained primary-level education (58.1%), were self-employed (62.0%), and were married (32.2%). Almost all participants (99.3%) initiated ART within 2 weeks of HIV diagnosis and were started on a fixed dose combination of Tenofovir Disoproxil Fumarate (TDF) + Lamivudine (3TC) + Dolutegravir (DTG), collectively abbreviated as TLD. Nearly two-thirds (63.5%) had WHO HIV clinical stage 1.

**Table 1:** Socio-demographic and clinical characteristics of HIV-infected treatment naïve individuals initiating ART, N = 430

Variable	number (%)
Sex	
Female	279 (64.9)
Site of recruitment	
TRRH	28 (6.5)
MRTH	264 (61.4)
MRRH	138 (32.1)
Level of education	
Informal	34 (7.9)
Primary level	250 (58.1)
Secondary level	114 (26.5)
University or college	32 (7.5)
Employment status, N = 429	
Employed	78 (18.2)
Self-employed	266 (62.0)
Unemployed	85 (19.8)
Marital status, N = 428	
Single	111 (25.9)
Married	138 (32.2)
Divorced	95 (22.2)
Cohabiting	47 (11.0)
Widowed	37 (8.6)
Duration from HIV Diagnosis to ART initiation, N = 426	
≤ 2 weeks	407(95.5)
> 2 weeks	19 (4.5)
WHO HIV Clinical stage, N = 427	
Stage 1	271 (63.5)
Stage 2	97 (22.7)
Stage 3	49 (11.5)
Stage 4	10 (2.3)
ART regimen initiated, N = 423	
TDF + 3TC + DTG	420 (99.3)
(TDF + 3TC + DTG) + DTG	2 (0.5)
TDF + FTC + ATV/r	1 (0.2)

Abbreviations: TDF = Tenofovir Disoproxil Fumarate; 3TC = Lamivudine; DTG = Dolutegravir; FTC = Emtricitabine; ATV/r = Atazanavir/ritonavir.

### Prevalence of HTN and risk factors for CVDs

The prevalence of HTN was 106/428 (24.8%) and that of pre-HTN was 158/428 (36.9%). The median (IQR) SBP and median (IQR) DBP were 123.00 (110.50, 134.38) mmHg and 74.5 (69.5, 81.5) mmHg respectively. The median TC (IQR), LDL-C (IQR), and TG (IQR) were 3.88 (3.06, 5.01) mmol/L, 2.36 (1.80, 3.06) mmol/L and 1.20 (0.90, 1.56) mmol/L respectively. The median HDL- C (IQR) was 1.00 (0.75, 1.23) mmol/L for women and 0.84 (0.61, 1.04) mmol/L for men. The most prevalent risk factor for CVDs in this population was dyslipidaemia found in 88.3% of the participants. Lower HDL-C was the most common dyslipidaemia having been seen in 77.3% of the participants followed by elevated TG (22.1%), elevated TC (21.9%), and elevated LDL-C (18.4%). Almost half of the participants (49.3%) consumed alcohol. The median (IQR) BMI was 22.3 (19.6, 25.9). Nineteen percent of the participants were overweight and 10.1% were obese. The proportion of participants with risky age for CVDs was 16.7%. History of past or current cigarette smoking was positive in 16.1% of the participants. The least common risk factors for CVDs were a family history of CVDs (14%), personal history of CVDs (3.3%), and DM (0.9%), Table 2.

**Table 2:** Prevalence of HTN and Risk factors for CVDs among HIV-infected treatment naïve individuals initiating ART, N = 430

Risk factor	number (%)
BP categories, N = 428	
^ a^Normal BP	167 (39.0)
^ b^Pre- elevated BP	165 (38.6)
^ c^Elevated BP	96 (22.4)
History of hypertension	
Yes	17 (4.0)
History of use antihypertensives	
Yes	2 (0.5)
Hypertension by definition, N = 428	
Yes	106 (24.8)
Pre-hypertension	158 (36.9)
No	164 (38.3)
Risky Age for CVD	
Yes	72 (16.7)
BMI, N = 426	
Underweight (BMI < 18.5 kg/m^2^)	72 (16.9)
Normal weight (BMI = (18.5 to 24.9 kg/m^2^))	230 (54.0)
Overweight (BMI = (25.0 to 29.9) kg/ m^2^)	81 (19.0)
Obesity (BMI ³ 30.0 kg/ m^2^)	43 (10.1)
Participant’s history of CVD	
Yes	14 (3.3)
Family history of CVD	
Yes	60 (14.0)
History of Diabetes Mellitus	
Yes	4 (0.9)
History of use of antidiabetics	
Yes	1 (0.2)
DM by definition	
Yes	4 (0.9)
Cigarette smoking, N = 429	
Ever smoked	69 (16.1)
Alcohol consumption	
Ever consumed	212 (49.3)
TC, N = 415	
Elevated (TC ³ 5.17 mmol/ L)	91 (21.9)
LDL-C, N = 163	
Elevated (LDL-C ³ 3.36 mmol/ L)	30 (18.4)
HDL-C, N = 163	
Lower (HDL-C < 1.03 mmol/ L for men or 1.29 mmol/ L < for women)	126 (77.3)
TG, N = 163	
Elevated (TG ³ 1.70 mmol/ L)	36 (22.1)
History of use of antidyslipidaemics	
Yes	1 (0.2)
Dyslipidaemia, N = 163	
Yes	144 (88.3)

Notes: ^a^ (SBP < 120 mmHg and DBP < 80 mmHg); ^b^ (SBP= (120–129) mmHg and/ or DBP = (80–89) mmHg); ^c^ (SBP ≥ 140 mmHg and/ or DBP ≥ 90 mmHg)

### Predictors of hypertension

In both univariable and multivariable analyses, compared to participants who were normal or underweight, participants with overweight or obesity had 60% more occurrence of HTN (aPR 1.60, 95% CI 1.16–2.21: p-value = 0.01). Compared to single participants, divorced or widowed participants had 131% more occurrence of HTN (aPR 2.31, 95% CI 1.29–4.14: p-value = 0.01) while participants who were married or cohabiting had 146% more occurrence of HTN, (aPR 2.24, 95% CI 1.42–4.26: p-value = 0.001). Compared to participants in WHO HIV Clinical Stage 1, participants in Stage 3 had a 58% reduced frequency of occurrence of HTN **(**aPR 0.42, 95% CI 0.18–0.97: p-value = 0.04) (Table 3).

**Table 3:** Predictors of hypertension among HIV infected treatment naïve individuals initiating ART.

		Univariable analysis			Multivariable analysis		
Predictor	number (%)	cPR	95% CI	p- value	aPR	95% CI	p- value
^a^Risky Age for CVDs							
Yes	21 (29.2)	1.22	0.82–1.83	0.33	-	-	-
No	85 (23.9)	1			-		
Sex							
Male	43 (28.5)	1.25	0.90–1.75	**0.19**	1.38	0.96–1.99	0.08
Female	63 (22.7)	1			-		
Marital status							
Divorced/Widowed	37 (28.0)	2.37	1.33–4.23	**0.003**	2.31	1.29–4.14	**0.01**
Married/Cohabiting	56 (30.4)	2.58	1.48–4.49	**0.001**	2.46	1.42–4.26	**0.001**
Single	13 (11.8)	1			1		
Level of education							
University/College	7 (21.9)	0.8	0.34–1.90	0.62	-	-	-
Secondary level	29 (25.4)	0.93	0.49–1.77	0.83	-	-	-
Primary level	61 (24.5)	0.9	0.49–1.63	0.73	-	-	-
Informal	9 (27.3)	1			-		
Alcohol consumption							
Ever consumed	52 (24.6)	0.99	0.71–1.38	0.95	-	-	-
Never consumed	54 (24.9)	1			-		
Cigarette smoking							
Ever smoked	14 (20.3)	0.79	0.48–1.31	0.36	-	-	-
Never smoked	92 (25.6)	1			-		
Duration from HIV Diagnosis to ART initiation							
> 2 weeks	2 (10.5)	0.41	0.11–1.54	**0.19**	0.41	0.11–1.50	0.18
≤ 2 weeks	104 (25.7)	1			1		
WHO HIV Clinical stage							
Stage 4	3 (30.0)	0.99	0.38–2.59	0.98	1.12	0.49–2.55	0.79
Stage 3	5 (10.2)	0.34	0.14–0.79	**0.01**	0.42	0.18–0.97	**0.04**
Stage 2	16 (16.7)	0.55	0.34–0.89	**0.02**	0.63	0.39–1.02	0.06
Stage 1	82 (30.4)	1			1		
DM							
Yes	2 (50.0)	2.04	0.75–5.51	**0.16**	2.13	0.60–7.59	0.25
No	104 (24.5)	1			1		
Family history of CVD							
Yes	18 (30.0)	1.26	0.82–1.92	0.3	-	-	-
No	88 (23.9)	1			-		
Dyslipidaemia							
Yes	47 (32.6)	1.24	0.56–2.73	0.59	-	-	-
No	5 (26.3)				-		
BMI							
Overweight/Obesity	44 (35.5)	1.72	1.24–2.38	**0.001**	1.6	1.16–2.21	**0.01**
Underweight/Normal weight	62 (20.7)	1			1		

Abbreviations: cPR = crude prevalence ratio; aPR = adjusted prevalence ratio; CI = confidence interval

Notes: ^a^ (male ≥ 45 years, female ≥ 55 years);

## Discussion

The present study presents data on the prevalence of HTN, traditional risk factors for CVDs, and their association among treatment naïve HIV-infected individuals initiating ART in Urban Tanzania. This was a cross-sectional analysis of data at the enrolment of 430 participants of a clinical trial aiming at determining the effect of low-dose aspirin on HIV disease progression among HIV-infected individuals initiating ART. The prevalence of HTN, the primary outcome, was 24.8%. Dyslipidaemia, alcohol consumption, and overweight/obesity were the most prevalent traditional risk factors for CVDs while a family history of CVDs, a personal history of CVDs, and DM were the least common. Being overweight or obese significantly predicted HTN while WHO HIV clinical stage 3 appeared protective against HTN.

About a quarter of our study participants had HTN. This is in keeping with findings from a study in Nigeria among ART naïve PLHIV [32] and the WHO African region and global prevalence of HTN among the general population [33]. However, studies conducted in Tanzania over five years ago reported a lower prevalence of HTN in ART naïve PLHIV in the same urban settings and one in rural settings, ranging from 5.3 to 12.5% [9, 34–37]. This may interpret as an increasing trend of HTN in the ART naïve population reflecting the similar trend of non-communicable diseases facing the general Tanzanian population.

The prevalence of HTN in this study is however higher than those previously reported in other parts of Africa, America, Europe, and Asia among ART naïve PLHIV [38–43] signifying that more research is needed to simultaneously correlate HTN burden among PLHIV and that among the general population in a locality.

The prevalence of HTN in the present study is alarmingly high and expected to be higher during chronic use of ART. This thought is derived from previously reported higher prevalence of HTN in ART exposed population that implicate ART as one of the causes of HTN in the PLHIV [39, 42, 44–48]. Specifically, Dolutegravir (DTG) based ART regimen among others have been associated with HTN [39, 49–53]. In recent years, most of countries including Tanzania have adopted DTG based regimens as first line treatment for PLHIV. In the current study, almost all participants were planned to be initiated on TLD, a regimen containing DTG. Therefore, a double risk may exist in such a population at the start of ART and may be associated with more HTN and CVDs in the future.

Despite the huge evidence of an association between ART and HTN in PLHIV, the prevalence of HTN in the current study is similar to or even higher than in the ART exposed [8, 34, 38, 49, 54–58]. This points out the role of other factors, apart from ART, such as chronic immune activation and HIV- related factors in the pathophysiology of HIV- associated HTN.

The findings of the present study show that being overweight or obese increases the risk for HTN. This is similar to earlier studies in Tanzania and elsewhere independent of ART status [32, 36, 49, 58]. In addition, being male or old have been associated with HTN in PLHIV. It is well known that being male, older age and overweight or obese are risk factors for HTN. Nevertheless, prevention strategies for HTN in the general population need to be intensified to achieve an overall low risk for HTN that will also benefit PLHIV.

WHO clinical stage 3 also was associated with a decreased risk for HTN in our study. This is in keeping with a previous study also conducted in Tanzania among ART naïve PLHIV [36]. At WHO clinical stage 3, ART naïve PLHIV have several opportunistic conditions and infections to present with overweight or obesity. In fact, one of the criteria to place PLHIV in WHO clinical stage 3 is severe unintentional body weight loss. Other known traditional risk factors for CVDs like DM, family history of CVDs, alcohol consumption, and cigarette smoking were not associated with HTN in the current study. These were assessed through self-reports by participants thus lacking objectivity.

The present study reports the prevalence of HTN and associated traditional risk factors for CVDs among participants of a clinical trial and thus the findings may not reflect the general picture among the ART naïve PLHIV as clinical trial participants are carefully selected.

## Conclusions

The prevalence of HTN is significantly high among PLHIV who are initiating ART. Known traditional risk factors for CVDs in the general population are also rampant among ART naïve PLHIV in urban Tanzania. Dyslipidaemia, a known precursor for atherosclerosis and a number of CVDs together with other CVDs risk factors need screening and intervention early on ART initiation.

## Electronic supplementary material

Below is the link to the electronic supplementary material.

Additional File 1: List of trial-prohibited medications

## Data Availability

All data generated or analyzed during this study are included in this published article [and its supplementary files].

## Abbreviations

AMI: Acute Myocardial Infarction
ART: Antiretroviral Therapy
BMI: Body Mass Index
BP: Blood Pressure
CRFs: Case Report Forms
CTCs: Care and Treatment Centers
CV: Cardiovascular
CVDs: Cardiovascular Diseases
DBP: Diastolic Blood Pressure
DM: Diabetes Mellitus
DTG: Dolutegravir
HDL-C: High-density lipoprotein cholesterol
HTN: Hypertension
IQR: Interquartile Range
LDL-C: Low-density lipoprotein Cholesterol
MDH: Management and Development for Health
MRRH: Mwananyamala Regional Referral Hospital
MRTH: Mbagala Rangi Tatu Hospital
PACTR: Pan African Clinical Trial Registry
MUHAS: Muhimbili University of Health and Allied Sciences
NIMR: National Institute for Medical Research
PLHIV: People Living with HIV and/or AIDS
SBP: Systolic Blood Pressure
SD: Standard Deviation
SPSS: Statistical Software for Social Sciences
SSA: Sub-Saharan Africa
TC: Total Cholesterol
TDF: Tenofovir Disoproxil Fumarate
TG: Triglycerides
TLD: Tenofovir Lamivudine Dolutegravir
TRRH: Temeke Regional Referral Hospital
WHO: World Health Organisation
