# Supplementary material for: Hypertension and traditional risk factors for cardiovascular diseases among treatment naïve HIV- infected adults initiating antiretroviral therapy in Urban Tanzania
Source: BMC Cardiovasc Disord. 2023 Jun 20;23:309. doi: 10.1186/s12872-023-03332-6 (PMC10283332; doi:10.1186/s12872-023-03332-6)
Supplement: Supplementary file 1 — Additional File 1: List of trial-prohibited medications [file 12872_2023_3332_MOESM1_ESM.docx]

Supplementary file 1– Prohibited drugs during the study (Also used for eligibility check)

**Anti-coagulants**: Warfarin, Acenocoumarol, Phenindione, Dabigatran, Unfractionated Heparin, Low molecular weight heparin^1^, Rivaroxaban, Apixaban, Argatroban, Fondaparinux,Edoxaban **Anti-platelets:** Clopidogrel, Dipyridamole, Prasugrel, Ticagrelor, Abciximab, Tirofiban, Eptifibatide, Epoprostenol

**LONG-TERM NSAIDS^2^:** Ibuprofen, Naproxen, Diclofenac, Acelofenac, Fenoprofen, Flurbiprofen, Ketoprofen, Dexketoprofen, Tiaprofenic acid, Etodolac, Indomethacin, Meloxicam, Tenoxicam, Nabumetone, Phenylbutazone, Ketorolac, Piroxican, Sulindac, Tolfenamic acid, Celecoxib, Etoricoxib

**Aspirin:** (including over-the-counter) Nu-seals, Anadin, Beechams powders, Alka-seltzer, Disprin,Codis500
**Others:** Methotrexate, Long-term corticosteroids^3^ (e.g. dexamethasone, prednisolone, hydrocortisone)

1. Low molecular weight heparin at a prophylactic dose for inpatient thromboembolism is permitted.

2. Non-steroidal anti-inflammatory drugs (NSAIDs) should be avoided wherever possible but short-term intermittent NSAID use is allowed. NSAIDs should not be co-administered with the trial treatment for more than two consecutive weeks). Paracetamol can be considered as an alternative analgesic and is permitted within the trial.

3. Short-term intermittent systemic corticosteroids are permitted however longer term use (longer than two continuous weeks) is not permitted.
